# On the Post-Processing of 3D-Printed ABS Parts

**DOI:** 10.3390/polym13101559

**Published:** 2021-05-13

**Authors:** Mohammad Reza Khosravani, Jonas Schüürmann, Filippo Berto, Tamara Reinicke

**Affiliations:** 1 Chair of Product Development, University of Siegen, Paul-Bonatz-Str. 9-11, 57068 Siegen, Germany; jonas.schueuermann@student.uni-siegen.de (J.S.); tamara.reinicke@uni-siegen.de (T.R.); 2 Department of Mechanical and Industrial Engineering, Norwegian University of Science and Technology (NTNU), 7491 Trondheim, Norway; filippo.berto@ntnu.no

**Keywords:** additive manufacturing, surface modification, roughness, mechanical properties

## Abstract

Application of Additive Manufacturing (AM) has significantly increased in the past few years. AM also known as three-dimensional (3D) printing has been currently used in fabrication of prototypes and end-use products. Considering the new applications of additively manufactured components, it is necessary to study structural details of these parts. In the current study, influence of a post-processing on the mechanical properties of 3D-printed parts has been investigated. To this aim, Acrylonitrile Butadiene Styrene (ABS) material was used to produce test coupons based on the Fused Deposition Modeling (FDM) process. More in deep, a device was designed and fabricated to fix imperfection and provide smooth surfaces on the 3D-printed ABS specimens. Later, original and treated specimens were subjected to a series of tensile loads, three-point bending tests, and water absorption tests. The experimental tests indicated fracture load in untreated dog-bone shaped specimen was 2026.1 N which was decreased to 1951.7 N after surface treatment. Moreover, the performed surface treatment was lead and decrease in tensile strength from 29.37 MPa to 26.25 MPa. Comparison of the results confirmed effects of the surface modification on the fracture toughness of the examined semi-circular bending components. Moreover, a 3D laser microscope was used for visual investigation of the specimens. The documented results are beneficial for next designs and optimization of finishing processes.

## 1. Introduction

Additive Manufacturing (AM) has been a prevalent technique for fabrication of various components. AM also knowns as three-dimensional (3D) printing is based on layer-by-layer manufacturing method. Unlike traditional manufacturing technologies involving forming (e.g., injection molding, bending, and stamping) and subtraction (e.g., shearing cutting), 3D printing joins materials together to make products. Compared to traditional manufacturing processes, AM indicated its advantages in energy saving, minimizing environmental impacts, and excellent scalability. More in deep, the main advantages of AM are reduction in waste materials, shortening the manufacturing cycle, minimizing cost of the low production runs, and fabrication of geometrically complex parts in a short period of time compared to traditional manufacturing processes. Considering potential benefits of AM, this manufacturing process has been used in different fields such as automotive, electronics, aerospace, healthcare monitoring, construction, and fashion [1,2,3,4,5,6]. Different AM processes have been used to print almost any item ranging from small size as toy and jewelry to the large size such as car and house [7].

3D printing has been extensively used for production of different structural components from ceramics [8], polymers [9], metals [10], glass [11], and multimaterials [12] through 3D models. Compared to the manufacturing processes which required machining, molds, and tooling, 3D printing is economically favorable and it is much more flexible. Moreover, in this manufacturing process design mistakes can be avoided by an evaluation at an earlier stage of product development. Although 3D printing presented aforementioned advantages, quality, mechanical behavior, and performance of 3D-printed parts depend on various parameters. In this context, different engineering aspects have been studied in this field [13,14,15,16,17,18]. For instance, developments of constitutive material models of the 3D-printed parts via FDM is presented in [19]. Indeed, the researchers emphasized on utilizing numerical homogenization procedure in the constitutive material model of additively manufactured parts. More in deep, they investigated effects of layer deposition with different raster orientation on the mechanical behavior of the printed part. The presented results can be used for stress analysis and selecting correct constitutive material model.

Although 3D printing was introduced as a manufacturing process for production of prototypes and low production runs, it has been utilized for fabrication of final products. Therefore, quality and mechanical behavior of these parts have become of significant importance. In this context, researchers investigated strength, structural integrity, and quality of the additively manufactured parts [20,21,22,23,24]. For instance, in [25] fracture theories were utilized for analyzing of 3D-printed biomimetic composites. To this aim, specimens were printed based on material jetting process and a series of compact tension tests was performed to determine fracture toughness based on the relevant standard. The achieved results confirmed that the material microstructure plays a crucial role in toughening composites. The reported results is beneficial for the microstructural design to obtain a favorable failure pattern. In a subsequent study [26], tensile strength of ABS composites was increased using filler loadings. In this respect, ABS filled with various loading of CuO reinforcement and experienced tensile loading. The obtained results showed that the tensile strength was increased up to 56%. Recently, lifetime of 3D-printed mold inserts was investigated in [27]. In detail, 3D printing was used to produce tool insert for injection molding. The 3D-printed mold inserts were subjected to the accelerated thermal ageing and used in the actual injection molding process. After a certain number of shot, cracks started and propagated until critical failure. The fractographic analysis was performed to determine influence of thermal ageing on crack initiation and lifetime of the mold inserts.

Based on the applications of the parts, in some cases a smooth surface of a 3D-printed part is required. In this context, several attempts are documented [28,29,30,31]. For instance, in [32] investigation was performed to determine effects of an aqueous NaOH treatment of 3D-printed PLA parts for surface activation. More in deep, PLA scaffolds were printed based on FDM process, and then treated with different NaOH solutions for different times in the range of 1 min to 48 h. Later, x-ray photoelectron spectroscopy measurements were carried out on the treated and untreated specimens. Moreover, the researchers claimed that there was no significant changes in mechanical properties of the tested specimens before and after treatment. In a recent study [33], applicability of 3D printing in the production of printed reinforcements has been confirmed. In this respect, 3D-printed mesh reinforced geopolymer was constructed and examined. The researchers produced NaOH solution by mixing sodium hydroxide with water and maintaining under specific conditions. The specimens experienced bending test conditions. Since chemical stability would be changed by treatment with NaOH, further investigation is required.

In [34], effects of sterilizations on the mechanical behavior of 3D-printed ABS parts were studied. As this condition might be occurred for medical instruments in a hospital environment, instrument functionality can be changed. However, the obtained results indicated that there is no significant differences between strength and stiffness of sterilized and non-sterilized specimens. Since ABS is a polymer that can be easily solved in acetone, it can be used for finishing of 3D-printed ABS parts for some applications. Therefore, it is necessary to determine influence of this post-processing on mechanical behavior of 3D-printed components.

In the current research, the authors experimentally investigated post-processing treatment of 3D-printed ABS components. To this aim, ABS material was utilized to print dog-bone shaped and semi-circular bending (SCB) specimens based on the FDM process. Later, acetone as colorless solvent was used in a post-processing to provide smooth surfaces in 3D-printed parts. Treated and untreated test coupons experienced tensile loads and three-point bending test and their mechanical behavior were compared. Moreover, the specimens were subjected to the water absorption tests and the obtained results were recorded. The reminder of this paper is organized as follows: in Section 2 details of FDM process and its parameters are briefly presented. Section 3 describes experimental procedures, which covers specimen fabrication, mechanical tests, and visual investigations. Finally, Section 4 concludes the paper.

## 2. Processing Parameters in FDM 3D Printing

**Figure 1:**
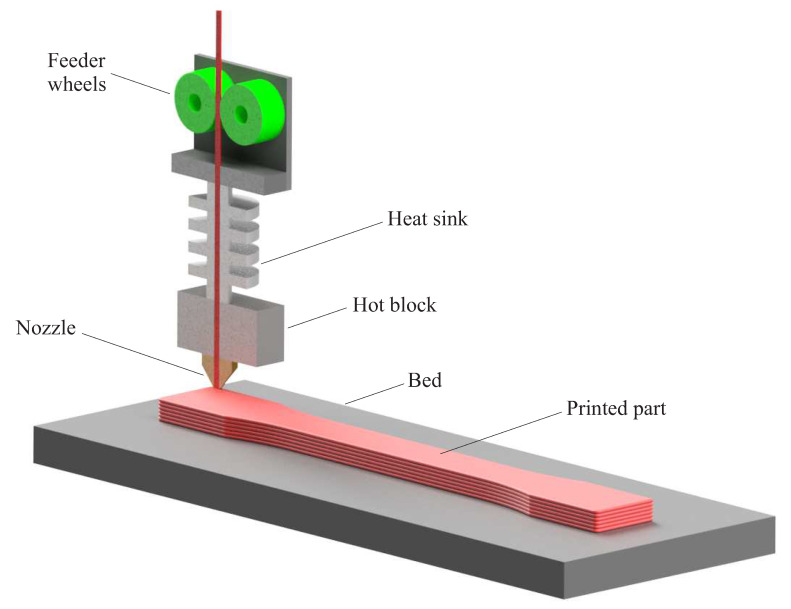
A schematic of the FDM printing process.

According to ASTM standard [35], 3D printing has been classified into seven classes: (a) sheet lamination, (b) binder jetting, (c) material extrusion, (d) direct energy deposition, (e) powder bed fusion, (f) material jetting, and (g) vat photopolymerization. Considering capacities and versatility, FDM is a popular method of 3D printing. The FDM is a low cost process based on the material extrusion through a nozzle that follows a predetermined path to print the component onto a previously printed layer or build plate in a layer-by-layer manner. The extruded material rapidly solidified and adhered with the surrounding material to build the required components. However, as FDM contains the deposition of subsequent layers achieved by the extrusion of molten materials, it is characterized by strong similarities with other hot-processing manufacturing technologies. Figure 1 shows a schematic of the FDM process. The FDM printing technology is well-known for fabrication of the structural elements utilizing filaments based on polymers with various characteristics. Indeed, polymeric material with different flexibility, working temperature, and hardness can be utilized. The most common materials used in the FDM are PLA, PC, and ABS. Moreover, in some researches short and long fiber reinforced thermal plastics were used as the feedstock of FDM process [36,37,38]. For instance, in [38] carbon fiber reinforced plastic composite specimens were printed based on the FDM using carbon fiber and ABS.

Although the FDM process has proved its advantages such as minimum wastage, low cost, and ease of material change, quality of the FDM printed parts depends on different parameters. In detail, final quality of the FDM 3D-printed parts depend on several factors that can be divided into three main stages: preparation, printing process, and post-processing. Here, we briefly elaborate some of the main factors in the aforementioned stages:

- Preparation factors: Design and geometry of model, file format and data transfer, and slicing (2D slicing, 3D slicing).
- Printing parameters: Nozzle temperature, nozzle diameter, bed temperature, raster direction, layer thickness, printing speed, raster width, and infill pattern.
- Post-processing: Separation technique and surface treatment.

It is noteworthy that different researches have been performed to determine influence of the above-mentioned factors on the quality and the performance of the 3D-printed parts. Moreover, several recommendations have been documented for different aforementioned stages. For instance, in [39] plating process was reviewed as a post-processing for FDM 3D-printed parts.

## 3. Experimental Procedure

### 3.1. Specimen Preparation

ABS material was used to fabricate the specimens. ABS is a theromplastic material which easily react with chemical finishing agents. Here, three groups of samples were fabricated with different geometries: (a) dog-bone shaped, (b) SCB test coupons, and (c) squar-shaped specimens based on the FDM process. The utilized ABS material parameters and printing process parameters for the first group of the above-mentioned specimens are summarized in Table 1. In the current study, ABS material from polymaker was used. A list of material parameter used in this investigation is available in [40].

**Figure 2:**
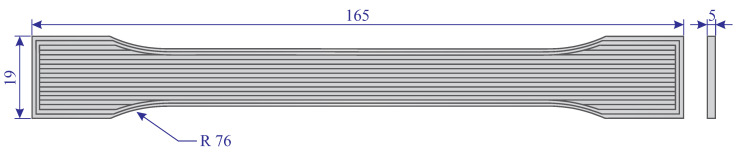
A schematic and dimensions of the dog-bone shaped specimen (dimensions in mm).

The dog-bone shaped specimens were designed according to ASTM D638 [41] for tensile tests. A schematic of a dog-bone shaped specimen and its dimensions are shown in Figure 2. In this study, we have printed six dog-bone shaped specimens and half of the specimens experienced surface modification as a post-processing treatment.

Moreover, we have designed SCB and squar-shaped specimens for three-point bending water absorption tests, respectively. The same ABS material was used for all specimens. Similar to the tensile test specimens, half of the SCB and squar-shaped specimens experienced surface treatment. Later, untreated and treated squar-shaped samples experienced water absorption tests to investigated effects of the surface treatment on the water absorption of the specimens.

### 3.2. Surface Treatment

**Figure 3:**
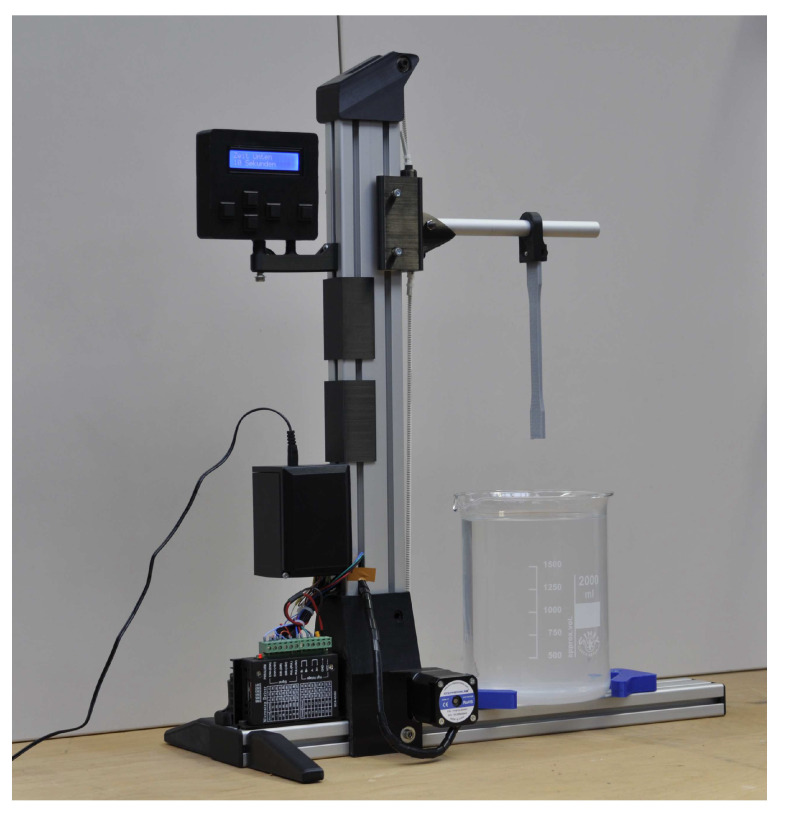
Fabricated equipment for surface treatment of 3D-printed parts.

In the current study, we have designed an fabricated a mechanical equipment to perform chemical surface treatment. The device includes vertical and horizontal axis (aluminum profile), 3D-printed guide blocks, belt drive, a glass contaider, a stepper motor, and a microprocessor. A stepper motor was selected, because of its sparkless operation. The microprocesor used 16 × 2 character LCD display to show the menu. There are two end stops on the axis and the dwell time is controlled by the processor. Figure 3 shows the fabricated device and dog-bone shaped specimen.

In the chemical treatment, 3D-printed part is immersed into the aceton solution for the selected time. The horizontal axis moved upward to remove the treated part. It is noteworthy that experimental practice shows that the surface treatment with acetone at different times clearly have different infleuneces on the surface quality. In detail, inapproperiate time for immersion of the specimen in acetone can make the surface quality even worse. Therefore, immersion time should be considered as an important factor which must be determined precisely. It depends on several parameters. For instance, type of material, complexity of component, and thickness of the specimen play important roles on the immersion time. In the current study, based on trial experiments, we have found that immersion for five seconds and repeating for two times with twenty seconds interval, leads to best results in the surface treatment of the examined 3D-printed ABS parts. It should be emphasized that immerssion into acetone has some advantages compared to surface treatment with vapour. For example, harmful gasses and fumes are not produced by imersion of sample. Moreover, imemersion into acetone solution is cheaper and faster compared to the hot vapour treatment.

### 3.3. Tensile Tests

**Figure 4:**
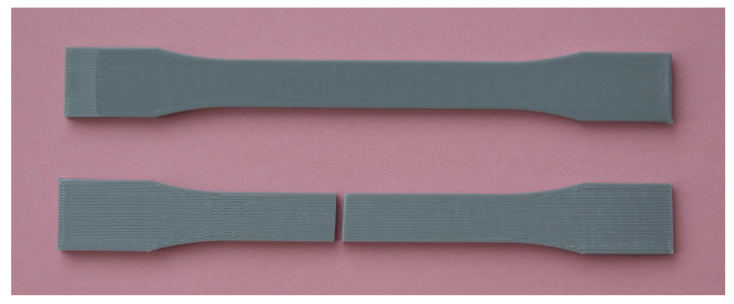
Dog-bone shaped specimens before and after tensile test.

A series of tensile tests was performed using a hydraulic test machine. In detail, treated and untreated specimens were subjected to tensile loads under quasi-static conditions at room temperature. The utilized machine was fitted with 15 kN load cell, with a cross-head speed in the range of 0.01 mm/s to 30 mm/s. Moreover, the tensile machine was equipped with an electronic control unit that provides monitoring of the applied load and movement of the top cross-head. In this study, specimens were subjected to mode I loading and the tests are conducted under displacement control via constant cross-head movement of 5 mm/min. Here, a series of tensile tests was performed according to [41]. The same test conditions were applied for untreated and treated specimens. Figure 4 shows the specimens before and after tensile test.

**Figure 5:**
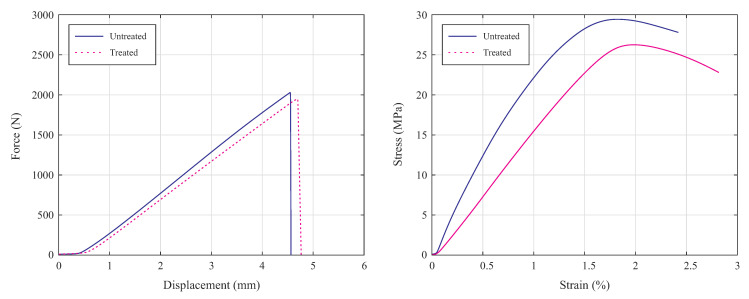
Force-displacement (**left**), and stress-strain curves of untreated and treated specimens (**right**).

It is noteworthy that in both groups of untreated and treated specimens, fractures were occurred in the gauge length of the specimens. This issue conformed that the obtained breaking strength is accurately represent the actual breaking strength of the specimens. The experimental practice indicated that the average fracture load of untreated specimens is 2026.1 N, while this value is deceased to 1951.7 N for the treated specimens. The average of force-displacement and tensile stress-strain curves of untreated and treated specimens are illustrated in Figure 5.

The experimental finding indicate that the tensile strength and Young’s modulus of the untreated specimens were higher than those of the treated ones. In detail, as can be seen in Figure 5, the maximum tensile strength of untreated specimen was 29.37 MPa which was reduced to 26.25 MPa in the acetone-treated specimens. Moreover, slope of the stress-strain curve was decreased in the treated specimens that shows reduction in Young’s modulus. During the chemical treatment with acetone solution, the outer surface of 3D-printed ABS specimens absorbs the chemicals, which will soften the material and it leads to a lower tensile strength. Indeed, the performed surface treatment has led to higher ductility and polished the surface texture, but to some extent the inner layers of 3D-printed parts also absorb the acetone which leads to reduction in tensile strength.

### 3.4. Three-Point Bending Tests

**Figure 6:**
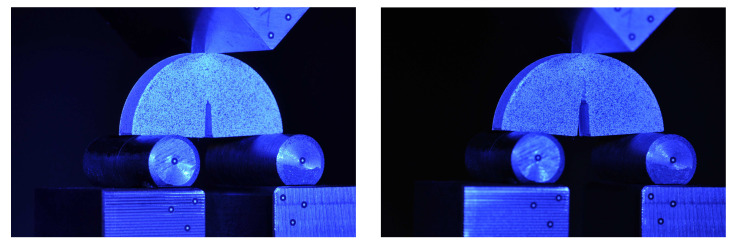
The SCB specimen under three-point bending test conditions; before loading (**left**), and after loading (**right**).

Three-point bending test has become popular because of its advantages. Experimental practice on SCB has been widely used to fracture toughness under different mode of failure. Indeed, as the SCB test uses a through-thickness crack, there is possibility to obtain stress intensity factor for pure mode I, mode II, or mixed-mode I/II conditions. Compared to other experimental investigations, tests on SCB specimens is relatively easy, special fixtures or jigs are not required, and this experimental setup can cover several failure modes. A frequent type of failure in engineering applications occurs as a result of the mode I loading conditions. In this case, the intensity of the near-tip crack fields is controlled by the stress intensity factor ($K_{I}$) in mode I. Based on the linear elastic fracture mechanics theory, the onset of fracture growth would be occurred when the stress intensity factor reaches the fracture toughness of the examined material, $K_{I}=K_{Ic}$. In the current study, we have conducted a series of tests on SCB 3D-printed specimens. In this context, both groups of untreated and treated specimens were examined. Figure 6 shows a SCB specimen under test conditions.

**Figure 7:**
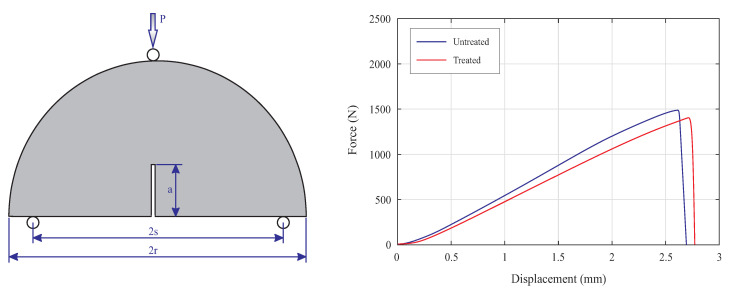
The SCB specimen geometry and size (**left**), and force-displacement curve of untreated and treated specimens (**right**).

In the current study, the SCB specimens were subjected to the vertical loading and tests were performed under displacement control condition via a constant cross-head movement of 1 mm/min. Specimen size and geometry and average of force-displacement curves of the examined specimens are illustrated in Figure 7. The SCB specimens were designed and printed with diameter (2r) of 60 mm and thickness of 10 mm. As can be seen in Figure 7, there is a crack in the middle of the SCB specimens with length (*a*) and width of 13 mm and 1 mm, respectively. In the SCB specimen, the notch length should be such that 0.4 ≤a/R≤0.6, where *a* and *R* are crack length radius of the specimens, respectively. In the fabricated 3D-printed specimens, a/R = 0.43. Moreover, in the examined specimens there is no angle between the initial crack and loading direction. Therefore, specimens were experienced pure mode I. The load was applied until the final fracture and obtained results showed that the load-displacement curves for all specimens were linear until failure load.

Based on experimental results, we have documented average of fracture load ($P_{max}$) for untreated and treated specimens, 1493.4 N and 1425.5 N, respectively. Therefore, the applied surface treatment was led to reduction in the fracture load. This maximum load should be utilized to calculate fracture toughness. In mode I, the fracture toughness can be calculated by:

(1) $$K_{I}=\sigma_{max}Y_{I}\sqrt{\pi \;a}$$

where

(2) $$\sigma_{max}=\frac{P_{max}}{2\;rt}$$

and $\sigma_{max}$ is maximum applied stress. Based on different research works [42,43], $Y_{1}$ can be determined using following equations:

(3) $$Y_{1}=-1.297+9.516\left(s/2r\right)-\left(0.47+16.457\right)\left(s/2r\right)\beta +\left(1.071+34.401\left(s/2r\right)\right)\beta^{2}$$

and

(4) $$Y_{1}=4.782-1.219\left(a/r\right)+0.063\;exp\left(7.045\left(a/r\right)\right)$$

where *t* and *s* are thickness of the SCB specimen and half of the distance between two supports on the experimental setup. Based on sung Equation (3), fracture toughness of untreated and treated specimens are 2.62 MPa$\sqrt{m}$ and 2.49 MPa$\sqrt{m}$, respectively. Utilizing Equation (4) has led to fracture toughness equal to 2.79 MPa$\sqrt{m}$ in untreated specimens. This value was reduced to 2.67 MPa$\sqrt{m}$ in treated specimens. The calculated values confirmed that the performed surface treatment has led to reduction in the fracture toughness of the 3D-printed ABS parts.

Based on the conducted tests and obtained results we concluded that the performed surface treatment and immersion in acetone solution increased ductility of the examined parts. As discussed in [44,45] different reasons such as molecular degradation and scission in molecular chains can be considered for reduction of resistance in polymeric materials. In the current study, the secondary bond between the ABS polymer chains breaks down. Similar to the dog-bone shaped specimens, reduction in fracture toughness of SCB specimens could be due to the absorption of acetone by the layers below the surface layer and the presence of acetone weakens the subsurface region.

### 3.5. Water Absorption Tests

In this study, water absorption tests were carried out based on ASTM D570 [46]. The squared-shaped specimens were used and a series of water absorption tests were performed with both untreated and treated specimens. In detail, the specimens were weighted before water immersion and the dry masses were documented. The water immersion test was performed on a glass container. In order to guarantee that water reaches the bottom part of the specimens, we used an absorbing filter paper placed at the bottom of the test coupons. Here, twenty-four hour immersion was conducted at temperature of 23 ± 1 ${}^{\circ }$C and at the end of the test, all the specimens are removed from the water one at a time, regarding to the standard procedure. In each defined time interval, the specimens were taken from the container, the excessive water was removed by a paper, weighted to the nearest 0.001 g immediately, and then replaced in the water. According to the Equation (5), percentage increase in weight during water immersion can be calculated:

(5) $$\mathrm{Increase}\mathrm{in}\mathrm{weight}\;\left(\%\right)=\frac{\mathrm{wet}\mathrm{weight}-\mathrm{conditioned}\mathrm{weight}}{\mathrm{conditioned}\mathrm{weight}}\times 100$$

where wet weight denotes the mass at he end of the experiment. Also, conditioned weight refers to the mass at the beginning of the test. The difference between the substantially saturated weight and the dry weight should be considered as the water absorbed when substantially saturated. The weighing of the specimens was performed in intervals of 15 ± 1 min for the first one hour, in intervals of 30 ± 1 min for the next four hours, and in intervals of one hour till end of the test. The documented weights indicated that the weight gain per unit area of the specimen was reduced from 2.12 × 10${}^{-3}$ g·cm${}^{-2}$ in untreated specimens to 1.82 × 10${}^{-3}$ g·cm${}^{-2}$ in treated specimens. It means that the performed surface modification helps on preventing the absorption of water by 3D-printed ABS specimens. The variation from untreated to treated parts represents a reduction of water absorption of 15% in examined specimens. Based on the water absorption tests, the open porosoity (P) can be calculated by Equation (6):

(6) $$P=\frac{\mathrm{wet}\mathrm{weight}-\mathrm{conditioned}\mathrm{weight}/\rho H_{2}O}{\mathrm{conditioned}\mathrm{weight}}\times 100$$

where $\rho H_{2}O$ is the density of water. According to the recorded weights, the surface treated has led to a reduction in open porosity. The absorption coefficient can be calculated with respect to the square rot of the immersion time, and the weight gain per unit area of the specimen. The value of the water absorption coefficient indicates the time needed to absorb a volume unite of water. The surface modification of 3D-printed parts leads to an increase in value of the water absorption coefficient.

### 3.6. Visual Inspections

**Figure 8:**
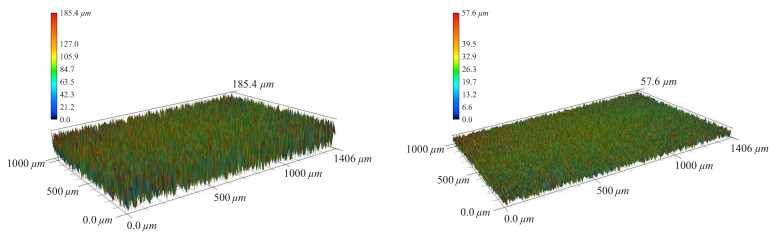
Surface of untreated (**left**), and treated (**right**) specimens.

In order to investigate the surfaces of the specimens before and after surface modification, a 3D laser microscope (KEYENCE VK 9700) was used. A measurement of a scan area of 2.07 × 1.55 mm${}^{2}$ was performed at three random positions of each specimen. Figure 8 represents the 3D topography of the 3D-printed ABS specimens before and after surface treatment. As can be seen in Figure 8, the surface roughness was reduced after the treatment process. Visual inspection of the specimens before and after surface treatment proved that the some defects such as small voids and gaps have been completely detached after the surface modification as a post-processing treatment. As a result of the conducted surface treatment, a slight flow of thermoplastic layer fills the gaps between the nearby layers. It has been led to improvement in surface quality.

The visual inspection confirmed that the surface unevenness almost vanished after the chemical treatment. The microscopic investigation showed that the average surface roughness of the specimens was reduced from 185.4 μm to 57.6 μm after surface treatment. Moreover, visual inspection was performed on the fracture surface of the dog-bone shaped specimens. This investigation indicated that although Infill percentage was set to 100% in the printed setup and it means the raster to raster air gap should be zero, there are still gaps between the filaments. The gaps confirmed that these slight air gaps are an intrinsic feature of the 3D-printed components. Moreover, visual investigation of the fractured specimens (which experienced mode I loading) indicated that there are only minor damage zones in the specimens.

## 4. Conclusions

Despite numerous applications of 3D printing in recent years, further research and development are required in several topics. As FDM process is based on the layer-by-layer deposition, FDM 3D-printed parts have typically low surface quality. Although 3D printing was used for prototyping, currently it is being utilized in rapid manufacturing of end-use products. Considering application of the part, sometime the end users are not satisfied with the surface quality of the 3D-printed components. Therefore, improving surface quality of FDM 3D-printed parts is a necessity. The present study reports application of acetone in a post-processing of 3D-printed components. In detail, ABS material was used for fabrication of parts based on the FDM process. Two groups of specimen (dog-bone and square-shaped specimens) were printed with the same printing parameters. Later, surface quality enhancement was performed by using a manufactured equipment. This device was designed and fabricated to use acetone in order to increase surface quality of 3D-printed parts. Untreated and treated specimens were subjected to the tensile load and water absorption tests. A series of tensile tests showed brittle failure in a normal direction to the tensile stress for untreated specimens. Immersion in acetone solution increased ductility in 3D-printed ABS parts. Comparison of the results indicated effects of the surface modification on the mechanical properties and performance of the examined parts. Based on the obtained results, fracture load and tensile strength of all 3D-printed ABS specimens were decreased after surface treatment. More in deep, in the dog-bone shaped parts average fracture load of untreated specimens was 2026.1 N which was reduced to 1951.7 N in the treated specimens. Moreover, in this study experimental practices indicated that tensile strength was 29.37 MPa and 26.25 MPa for untreated and treated specimens, respectively. The reduction in the tensile strength can be related to the softening of the material due to the absorbing the chemical during the chemical treatment. It is concluded that although acetone was used for polishing outer surface of the printed parts, the inner layers also absorb the acetone which have contributed in reduction of the tensile strength. Experiments on semi-circular specimens indicated decrease in fracture toughness of the examined parts. Particularly, fracture toughness of untreated specimens was 2.62 MPa$\sqrt{m}$ and it was reduced to 2.49 MPa$\sqrt{m}$ in the treated specimens. Moreover, surfaces of the specimens were investigated by a 3D laser microscope in order to measure their roughness before and after treatment. Since ABS easily reacts with chemical agents, the proposed method for surface treatment can be considered as a simple technique to make a smooth outer layer which is necessary for some structural elements. The reported results confirmed that the utilized method is an efficient method for surface modification of 3D-printed ABS parts, but reduction in tensile strength must be considered. In this case, a judgment is required in balancing between the strength and surface roughness of 3D-printed parts.

## Figures and Tables

**Table 1:** Processing parameters of dog-bone shaped 3D-printed specimens.

Material Parameters	Values	Printing Parameters	Values
Glass transition Temperature (${}^{\circ }$C)	101	Nozzle temperature (${}^{\circ }$C)	255
Charpy impact strength (kJ/m${}^{2}$)	12.6 ± 1.1	Printing speed (mm/s)	30
Softening temperature (${}^{\circ }$C)	104	Layer thickness (mm)	0.2
Bending modulus (MPa)	1339 ± 238	Bed temperature (${}^{\circ }$C)	100
Bending strength (MPa)	59.0 ± 1.3	Number of contours	2
Elongation at break (%)	2.7 ± 0.4	Infill percentage (%)	100
Melt index (g/10 min)	9–14	Number of layers	25
Density (gr/m${}^{3}$)	1.12	Raster angle (${}^{\circ }$)	0

## Data Availability

The data presented in this study are available on request from the corresponding author.
